# Effective Training Data Extraction Method to Improve Influenza Outbreak Prediction from Online News Articles: Deep Learning Model Study

**DOI:** 10.2196/23305

**Published:** 2021-05-25

**Authors:** Beakcheol Jang, Inhwan Kim, Jong Wook Kim

**Affiliations:** 1 Graduate School of Information Yonsei University Seoul Republic of Korea; 2 Department of Computer Science Sangmyung Univerisity Seoul Republic of Korea

**Keywords:** influenza, training data extraction, keyword, sorting, word embedding, Pearson correlation coefficient, long short-term memory, surveillance, infodemiology, infoveillance, model

## Abstract

**Background:**

Each year, influenza affects 3 to 5 million people and causes 290,000 to 650,000 fatalities worldwide. To reduce the fatalities caused by influenza, several countries have established influenza surveillance systems to collect early warning data. However, proper and timely warnings are hindered by a 1- to 2-week delay between the actual disease outbreaks and the publication of surveillance data. To address the issue, novel methods for influenza surveillance and prediction using real-time internet data (such as search queries, microblogging, and news) have been proposed. Some of the currently popular approaches extract online data and use machine learning to predict influenza occurrences in a classification mode. However, many of these methods extract training data subjectively, and it is difficult to capture the latent characteristics of the data correctly. There is a critical need to devise new approaches that focus on extracting training data by reflecting the latent characteristics of the data.

**Objective:**

In this paper, we propose an effective method to extract training data in a manner that reflects the hidden features and improves the performance by filtering and selecting only the keywords related to influenza before the prediction.

**Methods:**

Although word embedding provides a distributed representation of words by encoding the hidden relationships between various tokens, we enhanced the word embeddings by selecting keywords related to the influenza outbreak and sorting the extracted keywords using the Pearson correlation coefficient in order to solely keep the tokens with high correlation with the actual influenza outbreak. The keyword extraction process was followed by a predictive model based on long short-term memory that predicts the influenza outbreak. To assess the performance of the proposed predictive model, we used and compared a variety of word embedding techniques.

**Results:**

Word embedding without our proposed sorting process showed 0.8705 prediction accuracy when 50.2 keywords were selected on average. Conversely, word embedding using our proposed sorting process showed 0.8868 prediction accuracy and an improvement in prediction accuracy of 12.6%, although smaller amounts of training data were selected, with only 20.6 keywords on average.

**Conclusions:**

The sorting stage empowers the embedding process, which improves the feature extraction process because it acts as a knowledge base for the prediction component. The model outperformed other current approaches that use flat extraction before prediction.

## Introduction

Influenza is a highly contagious disease that affects 3 to 5 million people and kills 290,000 to 650,000 worldwide each year [1]. To track and counter its effects, various countries have established influenza surveillance systems, such as the European Influenza Surveillance Scheme in Europe and the Centers for Disease Control and Prevention (CDC) in the United States. These mechanisms provide clinical data, such as physician visits with influenza-like illness (ILI). However, a proper extraction of actionable insights is hindered by a delay of approximately 2 weeks for such information to become available. To solve this problem, studies in the field of infodemiology [2,3] have been trying to gain novel and effective insights into diseases from internet-based data. Hence, various recent studies in infodemiology have attempted to deter this time delay to predict impending outbreaks by monitoring influenza in real time using cloud-sourced data, such as online news articles and social network services [4-9].

Key studies have been conducted on influenza prediction systems based on search queries, including Google Flu Trends [10,11], in which Google provided surveillance and prediction services for influenza using search queries [2,10,12-16]. Twitter has recently received significant attention as a potential source of data for the prediction of influenza outbreaks. The number of studies that leverage tweets to predict influenza has multiplied and they have achieved moderately accurate prediction accuracy [17-23]. The advantage of predicting a fast-spreading outbreak via social network data (such as Twitter) is the speed at which people can share the news, hence providing a prompt opportunity to use an analytical system to predict a serious outbreak. However, various obstacles—such as privacy issues for search query data—hinder the real-time prediction because of the failure to capture the inherent features of the data [24]. In addition, the tweets are created by amateur users and are prone to noise due to poor writing standards, typographical errors, use of jargon expressions, and meaningless content [19,25].

Previous studies have used these web data to surveil influenza outbreaks and improved predictive performance, but the problem exists that which data are used depends on the subjective choice of the experimenter [2,10,18,26,27]. Owing to these drawbacks, the performance of any machine learning approach that leverages such data depends on a meticulous extraction of data and the extraction of key latent features. Because training data are extracted from the internet based on keywords, it is important to select influenza-related keywords that perfectly reflect the latent characteristics of the data [10,18,26]. In previous studies, the keywords were selected by calculating the correlations between each word and influenza-related tokens [10], directly filtering all words that referred to influenza [19,25], or extracting all words that were subjectively related to influenza [27]. Calculating the correlations for all words is the most effective approach to selecting keywords that properly capture the hidden features of the data. However, this approach requires a lot of time because of the sheer number of correlation coefficients that must be calculated. On the other hand, the selection of keywords by screening the words that directly refer to influenza or are subjectively defined to be related to influenza fails to capture the ingrained features, even if the method is relatively fast.

To solve these problems, we proposed a method that combines word embedding [28-32] with cosine similarity to capture only the word vectors that are highly correlated with influenza using the distributed vectors. Filtering is followed by a sorting process that ranks these keywords according to their relationship with the actual influenza outbreak. To assess the effect of the sorting process on embeddings, we applied a long short-term memory (LSTM) [33] predictive model that predicts the impending influenza outbreak.

Word embedding is a natural language processing–based feature extraction technique that consists of establishing a distributed representation of words. Importantly, the features that are generated from word embedding can capture the context between tokens. However, in the context of influenza, using the features obtained through word embedding alone results in a large vector space that includes unnecessary tokens and deteriorates the prediction performance. To reduce the number of tokens to be considered in the prediction stage, the cosine similarity function empowers the word embedding by selecting influenza-related features according to their similarity.

After filtering the features of the tokens that are related to influenza keywords, it is also important to determine the optimal amount of training data to be used for the predictive model to improve its performance. To preferentially use keywords that are highly related to influenza outbreaks among keywords selected by word embedding and cosine similarity, these keywords are sorted using the Pearson correlation coefficient (PCC) [34] between the actual influenza outbreak keywords and the extracted features of the training data. The ultimate purpose of the sorting stage is to ensure that during the training, only the features that are highly correlated with the true features are input to the predictive model. The sorting reduces the error and facilitates the optimization process during the LSTM model training. The model is trained with the fine-grained features, and the sorting process improves the performance of the LSTM predictive model considerably. To assess the effect of the embedding process, various embedding approaches are evaluated.

We compared the model’s performance when the keywords used were sorted versus when they were unsorted. For the evaluation of the performance, we recorded the root-mean-square error (RMSE). FastText continuous bag-of-words (CBOW) outperformed other embedding schemes with a PCC of 0.8986 and an RMSE of 0.0090 with sorted keywords.

## Methods

### Online News Articles

Online news articles offer a rich opportunity to predict epidemic diseases such as influenza. However, news articles extracted based solely on the presence of the “influenza” token do not capture the hidden insights from the news. The main reason for this is the presence of noisy tokens, such as advertising content that has no association with influenza. To reflect the characteristics of the data, before keyword selection, we used an effective embedding stage to capture the latent relationship between words. Furthermore, to preferentially use the keywords most relevant to the influenza outbreak among the selected keywords, we sorted them according to the PCC based on the actual influenza outbreak and the proportion of news articles containing the keyword. Moreover, the classification model was trained on the extracted keywords.

### Main Components of the Overall Methodology

**Figure 1:**
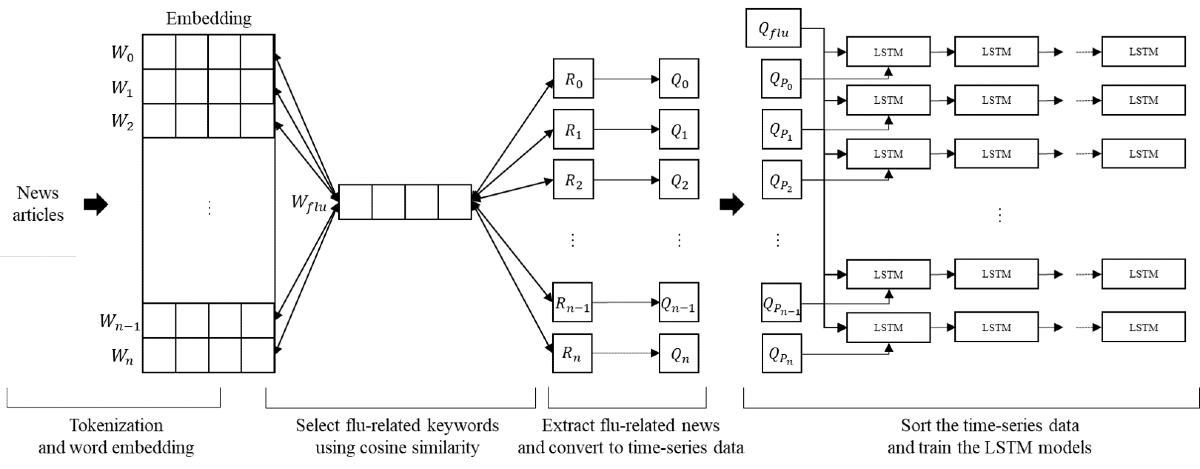
System architecture. LSTM: long short-term memory.

In this section, we cover the overall methodology, which includes 4 main parts: (1) tokenization and word embedding, (2) selection of flu-related keywords via cosine similarity, (3) extraction of flu-related news and its conversion into time-series data, and (4) training and classification. Figure 1 depicts the following 4 components of the model.

#### Tokenization and Word Embedding

Various tokens that are present in news articles do not have a semantic or syntactic relationship to the classification of the articles. Words such as “at” and “in” or adverbs such as “many” and “very” are filler words that must be removed before the embedding process. Hence, these stop words were stripped. To use only nouns as influenza-related words, tokenization was performed using the Mecab class provided by the morpheme analyzer KoNLPy [35]. The tokenized articles were fed to an embedding module that established a distributed representation of input tokens.

As shown in Figure 1, given the input article made of tokens 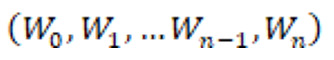, the objective of the embedding process is to learn a distributed feature representation of each token in the form of a distributed matrix, 
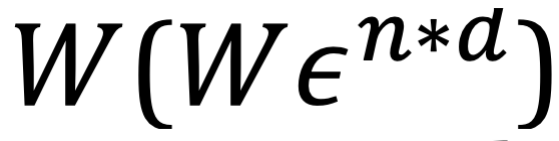
, where *n* represents the number of tokens and *d* represents the embedding size. The embedding matrix is structured such that the cosine similarity between the features that represent related tokens is higher. The generated vectors have the same dimension, and thus facilitate the training process.

Here, *W* learns a hidden vector that produces a context vector *W'* that considers other words when representing a given word. Given the input word, *W_i_*, the corresponding word vector in *W* (which is denoted as *v_wi_*) generates a corresponding context vector in *W'* (denoted as 
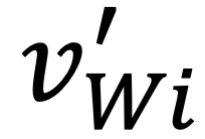
). The embedding output layer uses a softmax function to estimate the probability, 
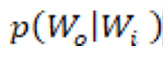
, of generating the output word *W_o_* from *W_i_* via the context vector as follows:

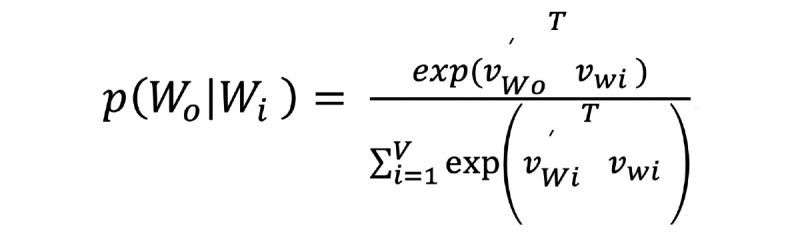



The vector assigned to each word uses the distance between the vectors to capture the relationship between words. Using the cosine similarity between the obtained embedding vectors, it is feasible to express the similarity between words. For instance, the results of the embedding are such that the cosine similarity between the vectors for “influenza” and “sneeze” is closer to 1 and is very close to the similarity between “malaria” and “fever.” Key hyperparameters are set during the training process. The embedding size *d* is the length of a dense vector that represents each word, and the window size is the number of words to be checked simultaneously to learn semantic relationships. The min count represents the minimum number of words to ponder during the training, and any word whose number of appearances is less than this count will be disregarded. In our implementation, we set the embedding size to 300, the window size to 5, and the min count to 100. There are various embedding approaches; in this study, we compared them to evaluate their performance in influenza detection. We compared Word2Vec skip-gram, Word2Vec CBOW, GloVe, FastText CBOW, and FastText skip-gram.

#### Selection of Flu-Related Keywords

The main objective of our model was to filter the influenza-related tokens to be considered for prediction. For this, we measured the cosine similarity to establish the closeness of each token with the word “influenza.” The cosine function was applied to the embeddings obtained in the previous step. Cosine similarity is a method of measuring the similarity between 2 vectors using the cosine between the 2 vectors. It has a value between –1 and 1. The formula to measure the similarity using vector *W* of a specific word and vector *W_flu_* of influenza is as follows:

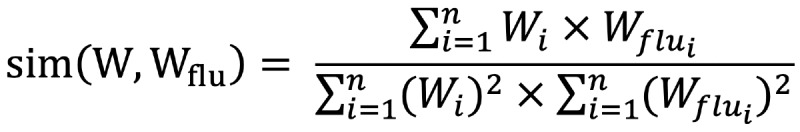



The above formula means that the inner product of vector *W* of a specific word and vector *W_flu_* of influenza is divided by the length of the 2 vectors. We selected *n* influenza-related keywords in the order of high cosine similarity.

#### Extraction of the Flu-Related News and Its Conversion Into Time-Series Data

Following the selection of influenza-related keywords, we extracted influenza-related news articles containing the keywords selected by word embedding and the word “influenza” simultaneously to ensure that the news articled reflected the characteristics of the data. In other words, news articles extracted through this process were a subset of the news articles that contained only the word “influenza.” The following step involved the conversion of news articles that contained only the word “influenza” and news articles that contained both the word “influenza” and the keywords selected by word embedding into time-series data to use as a training set. The *n* related keywords 
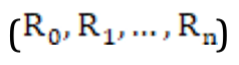
 selected by the word embeddings were converted into time-series data 
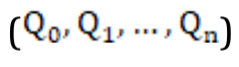
 by the following process:

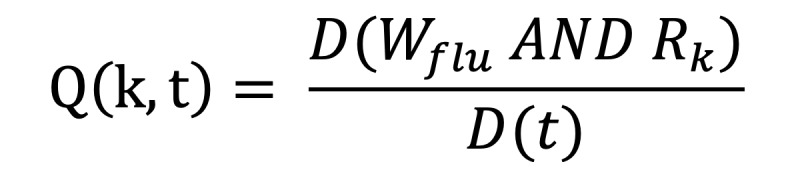



In the above equation, *D(t)* represents the number of news articles in the *t*-th week, and *D(W_flu_ AND R_k_)* is the number of news articles that contain both the word “influenza” and the related keyword *R_k_*. Therefore, Q(k, t) refers to the proportion of news articles containing both “influenza” and *R_k_* news articles from the *t*-th week. The time-series data Q_k_ are an array 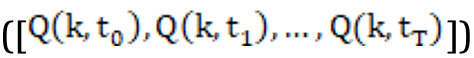 of Q(k, t) corresponding to each week 
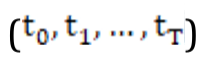
.

#### Sorting the Time-Series Data

Another key objective of the model was to capture a weekly match between influenza trends in news articles and the actual occurrences of influenza. Hence, the sorting of the obtained time-series data was critical to progressive prediction and trend capturing. Time-series data 
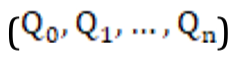
 extracted using the keyword selected by word embedding were in the order that was highly related to the word “influenza.” Therefore, we sorted the keywords and the time-series data based on the PCCs between the actual influenza outbreak and extracted time-series data to preferentially use the time-series data that were most relevant to the influenza outbreak. For example, since “headache” is a word associated with influenza symptoms that tends to appear alongside “influenza” in many news articles, the generated embeddings for these 2 tokens are likely to be close to encode a high association between “influenza” and “headache.” However, because “headache” is a symptom of various diseases, it can be difficult to determine if the “headache” in the text refers to “influenza” outbreak. Therefore, for effective training of influenza prediction, we applied a sorting process that preferentially uses highly relevant tokens to influenza outbreaks. After this step, we trained the (n+1) predictive model by adding the sorted time-series data 
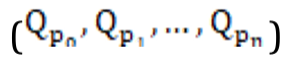
 sequentially to the time-series data extracted using only the word “influenza” (Q_flu_). This was performed to check the change in performance according to the additional training data and find the optimal number of training data. In other words, the input dimension of the *k*-th predictive model was k-1, and 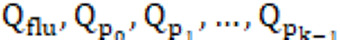 were used as training data.

#### Training of the Predictive LSTM Model

We built an LSTM model [33] to predict the weekly ILI-related cases. LSTM networks have recently been used for various prediction studies and performed well compared with vanilla recurrent neural networks (RNNs). LSTM networks use a gating mechanism that helps them overcome the vanishing gradient problem faced by RNNs. LSTM networks perform efficiently with time-series data, as they can choose which past information to forget or use while encoding a given time step. Bidirectional LSTM [36], recently studied in the field of natural language processing, showed better performance than unidirectional LSTM on average in time-series prediction such as influenza prediction [37]. However, in order to evaluate the proposed keyword selection process and the performance according to the type of word embeddings, we trained a prediction model using LSTM, which was mainly used in existing influenza studies [6,38,39].

During the training, we calculated the RMSE loss function, which is the square root of the difference between the predicted number of ILI cases and the actual numbers reported by the CDC. The model was optimized using the Adam optimizer [40], the time step was fixed to 5, and the layer size was set to 64.

## Results

### Embedding Models

To identify the most suitable word embeddings for the selection of influenza-related keywords, we selected 100 keywords that were highly related to influenza using 5 word-embedding models: Word2Vec CBOW, Word2Vec skip-gram, GloVe, FastText CBOW, and FastText skip-gram. The PCC [34] was used to sort the extracted keywords so that only the highly correlated ones were input to the LSTM model for training. The predictive accuracy of each model was evaluated using the PCC and the RMSE [41].

### Experimental Setup

We trained each word embedding model to evaluate its performance. As per the recent trend, many studies skip the embedding stage by using pretrained vectors. Although pretrained vectors are obtained from a large data set, they contain many tokens and have exhibited good performance in various recent studies. However, it is difficult to obtain efficient pretrained embeddings for languages other than English. Therefore, we collected approximately 2 million news articles over 2 years from September 11, 2017, to September 15, 2019, and the size of the collected data was approximately 761 MB, containing about 140,000 words as shown in Table 1. Table 2 shows the hyperparameters used when training word embeddings and the LSTM model. Epoch means the number of training repetitions; dimension of word embeddings means the dimension of the vector representing the word, and in the case of LSTM models it means the layer size. The window size of word embeddings means the number of surrounding words to be used for training, and min count means the minimum number of occurrences of words to be used for learning. The LSTM model's time step means how many weeks of data to use for prediction.

**Table 1:** Summary of news data for word embeddings.

Parameter	Value
Time period	September 11, 2017, to September 15, 2019
Total articles	2,093,120
Total bytes	761,233,009
Total terms	142,651

**Table 2:** Hyperparameters for word embeddings and long short-term memory model training.

Hyperparameter	Word embeddings	Long short-term memory model
Epoch	10	200
Dimension	300	64
Window size	5	–
Min count	100	–
Time step	–	5 weeks

### Experimental Results

**Figure 2:**
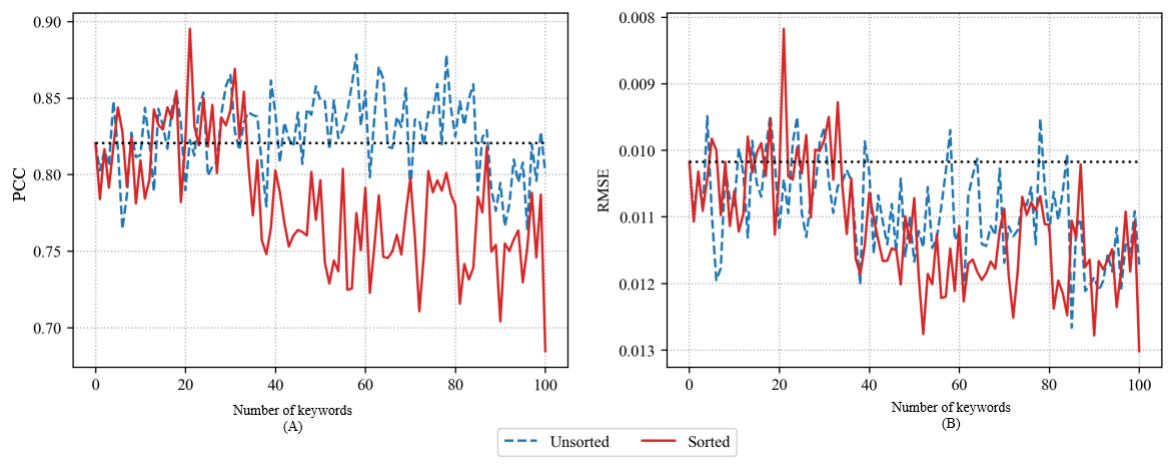
Pearson correlation coefficient (PCC) (A) and root-mean-square error (RMSE) (B) of long short-term memory models using Word2Vec continuous bag-of-words.

**Figure 3:**
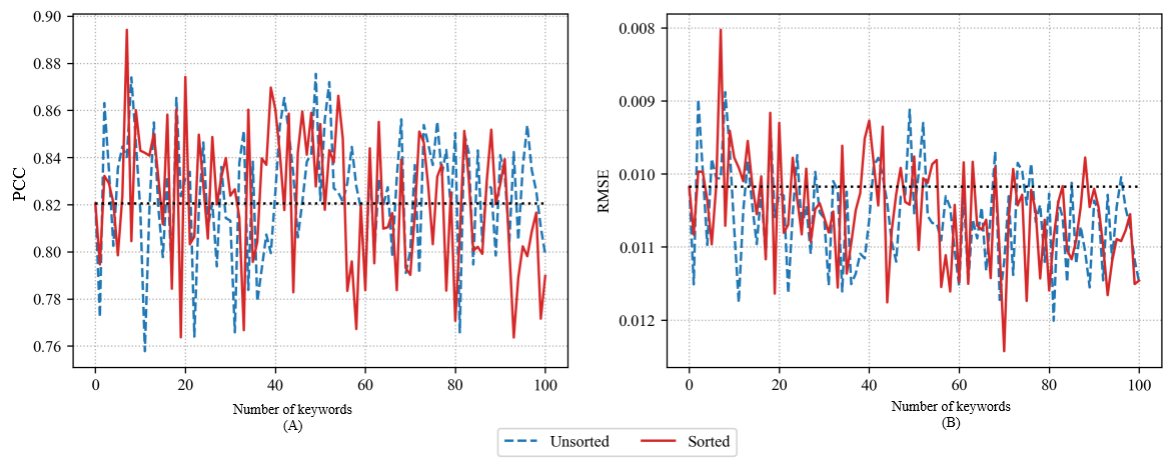
Pearson correlation coefficient (PCC) (A) and root-mean-square error (RMSE) (B) of long short-term memory models using Word2Vec skip-gram.

**Figure 4:**
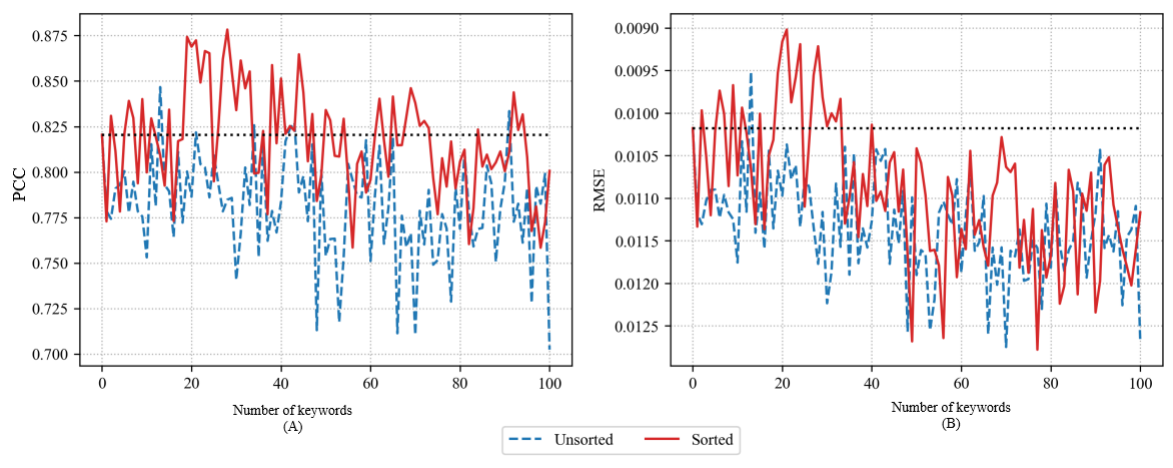
Pearson correlation coefficient (PCC) (A) and root-mean-square error (RMSE) (B) of long short-term memory models using GloVe.

**Figure 5:**
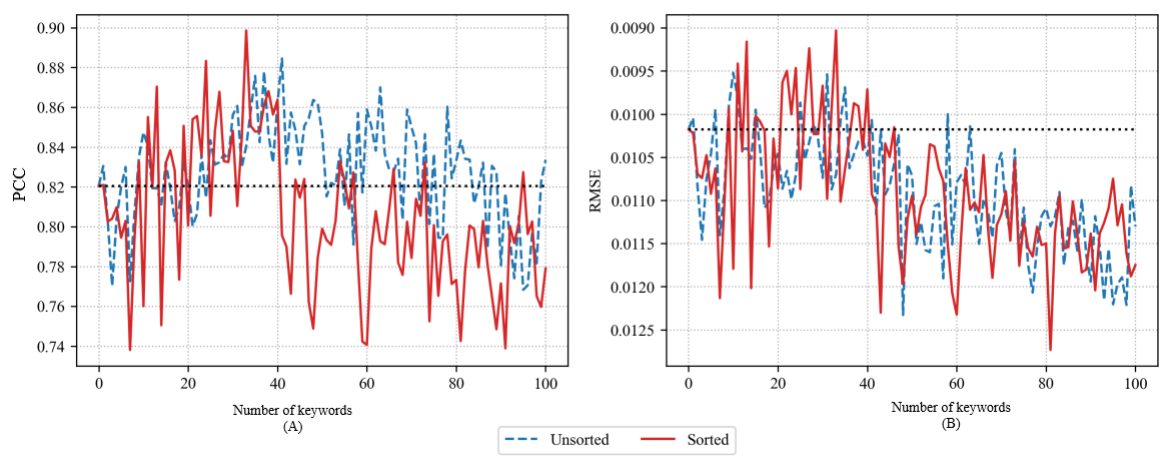
Pearson correlation coefficient (PCC) (A) and root-mean-square error (RMSE) (B) of long short-term memory models using FastText continuous bag-of-words.

**Figure 6:**
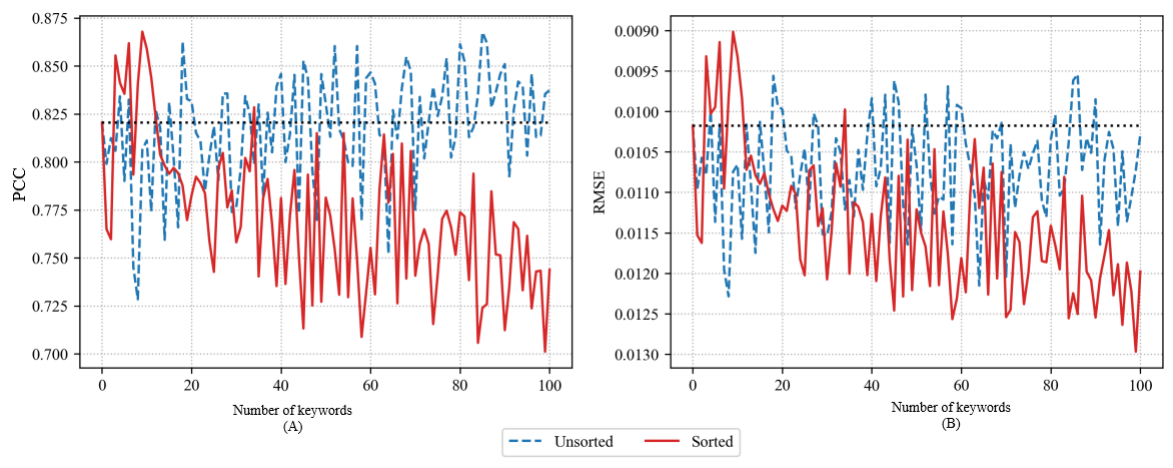
Pearson correlation coefficient (PCC) (A) and root-mean-square error (RMSE) (B) of long short-term memory models using FastText skip-gram.

Figures 2 to 6 show the accuracy of the predictive model for 100 keywords selected from each word embedding. The black dotted line in each figure depicts the condition when no keyword was selected and only “influenza” was used, and all time-series data related to the word “influenza” were used as input. Moreover, for each embedding schema, the figures show the PCC and the RMSE of the predictive model using the time-series data of only the word “influenza.” In the figures, “sorted” means that the keywords selected by the word embeddings were sorted based on the PCC—that is, the keywords were sorted in the order of their correlation with the influenza outbreak. “Unsorted” means that the keywords were not sorted. We expected that both sorted and unsorted approaches would show an accuracy increase to a certain level and then decrease with a further increase in the number of keywords. The sorted version achieved better accuracy than the unsorted method.

Figure 2 shows the accuracy of the LSTM model using PCC and RMSE when adding 1 to 100 time-series training data for the selected keyword using Word2Vec CBOW. As the number of keywords increased, both sorted and unsorted approaches showed an accuracy increase to a certain level and then decreased with a further increase in the number of keywords. The sorted version achieved better accuracy than the unsorted method. In the case of the sorted method, the maximum value achieved by PCC was 0.8951 with 22 keywords used, and the minimum RMSE value was 0.0082 when the same number of keywords was used. In the case of the unsorted method, the maximum PCC was 0.8784 with 59 keywords, and the minimum RMSE value was 0.0095 with 19 keywords. The sorted method showed better accuracy with fewer keywords. When using keywords that were highly related to influenza outbreaks, as the number of keywords increased, the accuracy decreased significantly. However, the decrease in accuracy was a natural result of using less relevant keywords. It was judged that the training data added in the sorted order had a more positive effect on accuracy improvement.

Figure 3 shows the accuracy of the LSTM model using PCC and RMSE when adding 1 to 100 time-series training data for the selected keyword using Word2Vec skip-gram. Both the sorted and unsorted methods of Word2Vec skip-gram showed repeated increases and decreases in accuracy as keywords were added. This means that the keywords selected using Word2Vec skip-gram were somewhat less related to the influenza outbreak than were the keywords selected using Word2Vec CBOW. However, in the case of the sorted method, although the repeated increase and decrease was large, it tended to increase to a certain level and then decrease with a further increase in the number of keywords. For the sorted keywords, the maximum PCC was 0.8942 with 8 keywords, and the minimum RMSE was 0.008 with the same number of keywords. In the case of the unsorted method, the maximum PCC was 0.8942 with 8 keywords, and the minimum RMSE was 0.0089 with 9 keywords.

Figure 4 shows the accuracy of the LSTM model using PCC and RMSE when adding 1 to 100 keywords using GloVe. The accuracy of the predictive model using GloVe was similar to that of the predictive model using Word2Vec CBOW. Both the unsorted and sorted methods temporarily exhibited a boost in accuracy as per the increase in the number of keywords. However, the accuracy gently decreased as the number of keywords increased further. Generally, the sorted method achieved higher accuracy. However, as shown in the figure, when the number of added keywords was very large, the accuracy of the unsorted and sorted methods was similar. In the case of the sorted method, the maximum PCC was 0.8783 with 29 keywords, and the minimum RMSE was 0.009 with 22 keywords. In the case of the unsorted method, the maximum PCC was 0.8467 with 14 keywords, and the minimum RMSE was 0.0095 with the same number of keywords.

The accuracy of the LSTM model using PCC and RMSE when adding 1 to 100 time-series training data for the selected keywords using FastText CBOW is depicted in Figure 5. Similar to the accuracy of the predictive model using the previous word embeddings, the sorted method outperformed the unsorted method. The sorted method achieved a maximum PCC of 0.8986 with 34 keywords and a minimum RMSE of 0.009 with the same number of keywords. The unsorted method achieved a maximum PCC of 0.8467 with 42 keywords and a minimum RMSE of 0.0095 with 11 keywords.

Figure 6 depicts the accuracy of the LSTM model using PCC and RMSE when adding 1 to 100 time-series training data for the selected keywords using FastText skip-gram. The general accuracy of unsorted and sorted methods was lower than that of other word embeddings covered thus far. This means that the time-series data for keywords selected using the FastText skip-gram were negatively correlated with actual influenza outbreaks. In the case of the sorted method, the maximum PCC was 0.8679 with 10 keywords, and the minimum RMSE was 0.009 with the same number of keywords. However, the model that used more keywords than the model with maximum accuracy showed a sharp decline in accuracy. The accuracy was lower than that of the model that used only “influenza” as a keyword. In the case of the unsorted method, the maximum PCC was 0.8676 with 86 keywords, and the minimum RMSE was 0.0095 with 87 keywords. However, similar to the sorted method, the accuracy increased sharply and decreased significantly.

### Analysis

In this study, we aimed to obtain the optimal word embedding when the PCC-based sorting was applied after keyword selection. We compared the best accuracy of the LSTM models trained using each type of word embedding against the number of selected keywords using PCC and RMSE. We considered 2 cases: whether PCC-based sorting was applied or not. Table 3 shows the highest accuracy of the LSTM predictive model using different word embedding techniques and the number of keywords used at each time. We found that the sorted method used fewer keywords but performed better on average. This means that using the data highly related to influenza outbreaks through the sorted method effectively selected training data and improved the average accuracy of the predictive model. Moreover, we found that among the word embedding techniques, FastText CBOW had the highest performance in terms of PCC and Word2Vec skip-gram had the highest performance in terms of RMSE. The process of training by using the context words is the same except that FastText produces a word vector using subword information while Word2Vec considers vectors for complete words. Therefore, there is a slight difference in the performance of Word2Vec and FastText, but it can be confirmed that they are very similar. GloVe, which utilizes the statistical data of the entire document, showed lower performance than the other embedding techniques.

**Table 3:** Pearson correlation coefficient (PCC) and root-mean-square error (RMSE) for influenza prediction models using different word embedding techniques.

Prediction model	PCC (number of keywords)		RMSE (number of keywords)	
	Unsorted	Sorted	Unsorted	Sorted
Word2Vec CBOW^a^	0.8784 (59)	0.8951 (22)	0.0095 (19)	0.0082 (22)
Word2Vec skip-gram	0.8755 (50)	0.8942 (8)	0.0089 (9)	0.0080 (8)
GloVe	0.8467 (14)	0.8783 (29)	0.0095 (14)	0.0090 (22)
FastText CBOW	0.8845 (42)	0.8986 (34)	0.0095 (11)	0.0090 (34)
FastText skip-gram	0.8676 (86)	0.8679 (10)	0.0095 (87)	0.0090 (10)
Mean	0.8705 (50)	0.8868 (21)	0.0094 (28)	0.0086 (19)

^a^CBOW: continuous bag-of-words.

**Figure 7:**
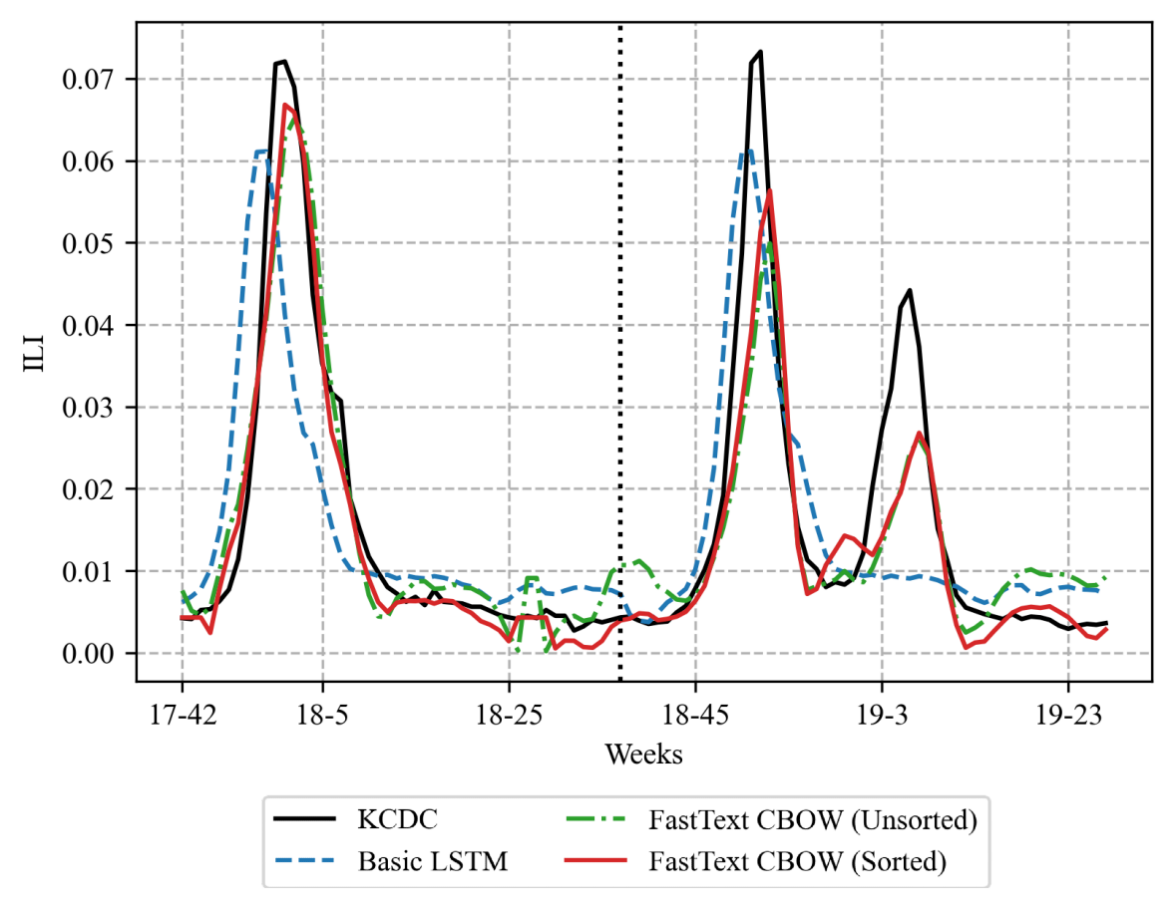
Comparison of actual influenza outbreaks and influenza prediction results from prediction models. CBOW: continuous bag-of-words; ILI: influenza-like illness; KCDC: Korea Centers for Disease Control and Prevention; LSTM: long short-term memory.

Figure 7 shows the prediction results of the model using only the time-series data of “influenza” (basic LSTM) and the unsorted and sorted methods using FastText CBOW, respectively, which showed the highest PCCs (Table 3). In Figure 7, the left side of the black dotted line drawn vertically at weeks 18-37 is the prediction result using the training data set, and the right side is the prediction result using the test data set. The predictive model using Korea Centers for Disease Control and Prevention ILI data and time-series data of only “influenza” hardly predicted the influenza peak at weeks 19-5 in the test data set. However, the predictive model trained on time-series data of additional keywords selected by FastText CBOW substantially improved the prediction accuracy compared with the model that used only the word “influenza.” In addition, the method that sorted the keywords selected by FastText CBOW based on PCC and added time-series data outperformed the unsorted method. Both unsorted and sorted methods using FastText CBOW predicted the influenza peaks at weeks 18-1 included in the training data set. However, neither method accurately predicted the influenza peaks at weeks 18-52 and 19-5 in the test data set. This is because the proportion of news articles containing the word “influenza” at the second (18-52) and the third (19-5) peak decreased compared with the first (18-1) peak, which affected the performance of all predictive models.

## Discussion

### Related Work

The accurate and timely prediction of influenza outbreaks has recently gained significant research attention. Many studies rely on legacy statistical approaches. High-performing methods use machine learning with internet-sourced and social network–sourced cloud data.

Eysenbach [2] found a close correlation between epidemiological data on flu and the number of clicks on Google's keyword-triggered links, which is based on the fact that many people use the internet to find health information. The PCC for the number of clicks in the current week and influenza cases in the following week was 0.91, which was a better predictor for influenza than ILIs reported by sentinel physicians. Eysenbach [2] also defined “information epidemiology” or “infodemiology” as a set of research methods such as tracking health information trends on the internet and distributing people's health information. Infodemiology data have the advantage that they can be collected and analyzed in real time.

Ginsberg et al [10] proposed a linear regression model using the search query from the Google search engine and the ILI data provided by the CDC in the United States to predict influenza. The rationale behind the study was that the search frequency of any influenza-related search query was correlated with the occurrence of influenza. The study established a list of candidate query groups to be used in the regression model by calculating the correlation between time-series forms of all search queries and the ILI value from the CDC. Hence, the top 100 of these correlated search queries were selected for training the model. The performance of the model improved depending on the number of highly correlated queries. The accuracy improved with 100 queries but did not improve with 45 queries.

Achrekar et al [19] proposed the framework of social network–enabled flu trends, which monitored flu trends. The study developed a model based on autoregression with an exogenous input that used tweets to predict influenza warnings and ILI occurrences. Tweets with the keywords “flu,” “H1N1,” and “swine flu” were defined as influenza-related tweets. Support vector machines (SVMs) [42] were used to exclude meaningless tweets. The study concluded that Twitter data were highly correlated with ILI rates.

Li and Cardie [25] developed a model that predicted influenza using Twitter and a probabilistic graphical Bayesian approach based on a Markov network. The approach divided influenza progression into 4 phases: nonepidemic, rising epidemic, stationary epidemic, and declining epidemic. Tweets containing the keywords “flu,” “H5N1,” “H5N9,” “swine flu,” and “bird flu” were defined as influenza-related tweets, and SVMs were used to remove the unnecessary tweets.

Zhang et al [27] implemented FluOutlook, an online system for predicting influenza outbreaks in 7 countries using statistical regression analysis and Global Epidemic and Mobility models [43,44]. The model was based on Influweb [45]—a voluntary participation information collection system—and Twitter. FluOutlook collected tweets containing 40-50 defined keywords and assigned a priority flag based on the correlation between the time-series data corresponding to each keyword and actual flu occurrences. The limited number of keywords helped mitigate the effect of noise included in the collected raw tweets.

These recent influenza prediction studies have used search queries and microblogging, such as Twitter, for real-time prediction. However, search queries provided by search engines (such as Google) cannot be used for real-time prediction because it is difficult and imprecise to infer the exact search trends. Moreover, as already asserted, Twitter and other social platforms are prone to noise. On the other hand, web-based news data exhibit less vulnerability to noise and have recently been adopted in several prediction studies [46-48]. The strength of these news data is due to real-time online accessibility and rigorous professional editing.

A crucial aspect to consider during the extraction of training data from the internet is the selection of keywords. Various studies calculated correlations for all words or used keywords that directly indicated influenza or were subjectively selected. Calculating the correlation coefficient for every token has been argued to be the best approach. However, it requires a lot of computing resources and training time. The direct or subjective selection of influenza-related keywords cannot be generalized to various data sets because it is challenging to extract the inherent features of the data set. Therefore, a method for selecting related keywords by reflecting the latent characteristics of the data during the selection of keywords improves the model considerably.

Various studies have also focused on word embedding as a feature extraction method that can capture the semantic and contextual aspects from texts by establishing a distributed representation of each token.

Mikolov et al [29,30] proposed Word2Vec—a model that uses a shallow neural network to assign a distributed vector to each word by calculating the co-occurrence probability. Using the distributional hypothesis [49], the probabilities are calculated such that words with close meaning or words that are likely to appear together in a certain context window are close in the vector space. The model consists of 2 distinct learning paradigms: skip-gram and CBOW. To build the distributed vector, skip-gram learns the probability of occurrence of context words from the target word, while CBOW learns the probability of occurrence of the target words from context words.

Word2Vec uses local information (context window) between words in the context by disregarding the global information. Hence, Pennington et al [31] proposed GloVe, which assigns a vector to each word by using the proportion of the target word appearing along with other words throughout the document.

Another key limitation of Word2Vec is that it ignores the internal morphology of words and fails to capture proper vectors for rare words. To address this limitation, Joulin et al [32] proposed FastText, which considers the subwords of each word. Rather than feeding the individual words to the neural network, FastText breaks them into n-grams and uses skip-grams to learn the distributed representation of each of these subwords. The final representation of a distinct word is the sum of these n-grams.

### Limitations and Future Work

When predicting influenza from news articles, we used word embedding to find words related to influenza and sorted them based on their association with actual influenza outbreaks, effectively extracting training data and improving the accuracy of predictions. However, our research has the following limitations, and future studies are needed. First, we need to check whether our approach works well for novel data sets other than news articles. Recently, influenza prediction has been studied using various data [38,50-53]. Therefore, it is necessary to study whether our approach can improve performance when applied to different data sets used in the recent state-of-the-art studies. In this study, we focused on improving the representation of the training data rather than on the learning scheme. Hence, we used the standard, unmodified LSTM model, which is widely used in existing influenza prediction studies [6,38,39]. However, research is being conducted to change the standard LSTM model in state-of-the-art influenza prediction [54,55] or to apply a prediction model that shows better performance in other fields [56,57]. Therefore, it is necessary to study whether our approach can lead to improvement in performance when applied to predictive models other than the standard LSTM model. Third, we used word embedding to extract keyword candidates for training data extraction, but we need to see if our sorting process can improve performance even when other keyword extraction methods are used.

### Conclusions

In this paper, we proposed an effective training data extraction method to improve influenza prediction from news articles. The input data selected by the extraction method encoded the relationship between the words with influenza-related keywords. Subsequently, these data were filtered as per their relationship with the actual influenza outbreak. This process was ensured by sorting the selected keywords based on PCCs between the actual influenza outbreak and the proportion of news articles containing the keywords. The predictive model that was trained on the extracted data using only the word “influenza” did not reflect the characteristics of the collected data; hence, it showed unsatisfactory performance. However, because the predictive models trained on the data extracted through the proposed method reflected the characteristics of the data, it was confirmed that the performance was greatly improved. We also compared the performance of the predictive models with 5 popular word embedding techniques. The experimental results proved that with the proposed method, FastText CBOW outperformed other embedding techniques with unsorted and sorted keywords.

## Abbreviations

CBOW: continuous bag-of-words
CDC: Centers for Disease Control and Prevention
ILI: influenza-like illness
LSTM: long short-term memory
PCC: Pearson correlation coefficient
RMSE: root-mean-square error
RNN: recurrent neural network
SVM: support vector machine
